# Organ structure and bacterial microbiogeography in a reproductive organ of the Hawaiian bobtail squid reveal dimensions of a defensive symbiosis

**DOI:** 10.1128/aem.02163-24

**Published:** 2025-04-15

**Authors:** Derrick L. Kamp, Allison H. Kerwin, Sarah J. McAnulty, Spencer V. Nyholm

**Affiliations:** 1 Department of Molecular and Cell Biology, University of Connecticut 21654 , Storrs, Connecticut, USA; 2 Department of Biology, McDaniel College 8541, Westminster, Maryland, USA; 3 Skype a Scientist, Philadelphia, Pennsylvania, USA; Norwegian University of Life Sciences, Ås, Norway

**Keywords:** symbiosis, accessory nidamental gland, microbiome, organ structure, spatial compartmentalization, microscopy, *Verrucomicrobia*, *Alphaproteobacteria*, *Gammaproteobacteria*, *Euprymna scolopes*

## Abstract

Many plants and animals house symbiotic microorganisms in specialized tissues or organs. Here, we used multidimensional *in situ* imaging techniques to illuminate how host organ structure and bacterial microbiogeography contribute to the symbiotic function of an organ in the Hawaiian bobtail squid, *Euprymna scolopes*. Along with the well-studied light organ, female *E. scolopes* harbor a community of bacteria in the accessory nidamental gland (ANG). The ANG is a dense network of epithelium-lined tubules, some of which are dominated by a single bacterial taxon. These bacteria are deposited into squid eggs, where they defend the developing embryos from harmful biofouling. This study used a combination of imaging techniques to visualize different dimensions of the ANG and its bacterial communities. Imaging entire organs with light sheet microscopy revealed that the ANG is a composite tissue of individual, non-intersecting tubules that each harbor their own bacterial population. The organ is bisected, with tubules converging toward two points at the posterior end. At these points, tubules empty into a space where bacteria can mix with squid jelly to be deposited onto eggs. Observations of the symbiotic community correlated bacterial taxa with cell morphology and revealed that tubule populations varied: some tubules contained populations of mixed taxa, whereas others contained only one bacterial genus. Together, these data shed light on how bacterial populations interact within the ANG and how the host uses physical structure to maintain and employ a symbiotic bacterial population in a defensive context.

**IMPORTANCE:**

Sequence-based microbiome studies have revealed much about how hosts interact with communities of symbiotic microbiota but often lack a spatial understanding of how microbes relate to each other and the host in which they reside. This study uses a combination of microscopy techniques to reveal how the structure of a symbiotic organ in the female bobtail squid, *Euprymna scolopes*, houses diverse, beneficial bacterial populations and deploys them for egg defense. These findings suggest that spatial partitioning may be key to harboring a diverse population of antimicrobial-producing bacteria and establishing a foundation for further understanding how host structures mediate symbiotic interactions.

## INTRODUCTION

“Form follows function” (1). A maxim originally associated with modern architecture can be applied to a host-microbe context: physical arrangements of organs and bacterial communities affect biology and inform our understanding of how they operate. Many organisms possess symbiotic organs that have evolved to recruit and maintain microbiota with diverse functions (2–4). Structural features of these organs facilitate contact with environmental microbes during recruitment (5, 6), allow symbiont entry into the host (7, 8), or sequester symbionts to specific areas to perform specialized functions (9–11). Disruption of this discreet localization of microbes can lead to dysbiosis and negative impacts on host health (12–15). The microbiogeography—the spatial arrangements the bacteria have in relation to each other and their host(s)—affects the microbial partners too: spatial fragmentation of microbial populations in “microniches” can reduce competition amongst strains (16, 17), allow specific strains to dominate an organ (18), and even drive the evolution of the bacterial community (19, 20). Furthermore, understanding the microbiogeography is vital to understanding the ecology and physiology of the entire system (21, 22).

**Fig 1:**
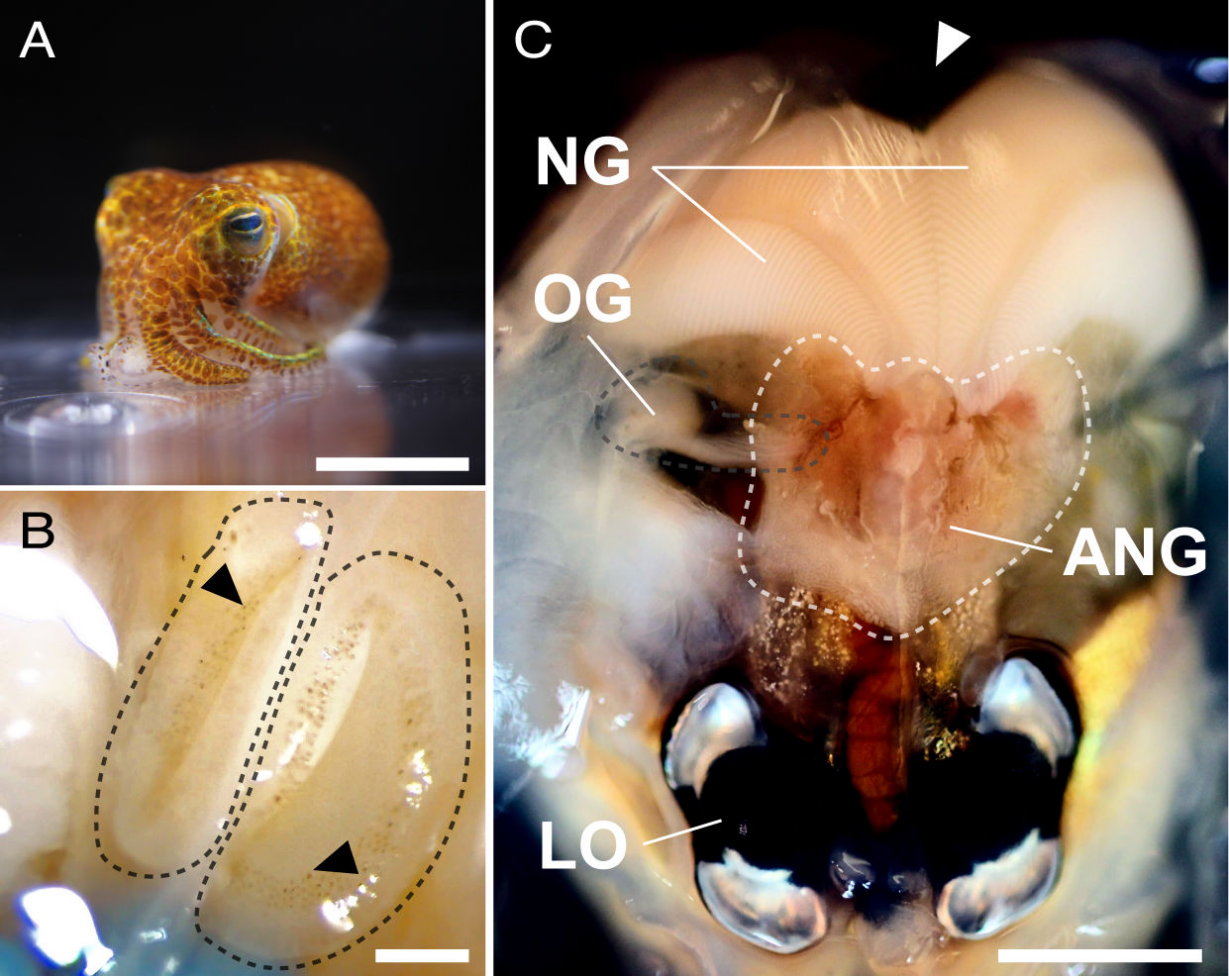
Anatomy of female *Euprymna scolopes*. (A) An adult Hawaiian bobtail squid, *E. scolopes*. (B) The early-stage ANG recruitment tissues. The two lobes of tissue (black outline) appear as “pads” approximately 4 weeks after the bobtail squid hatches and have ciliated pores (black arrowheads) that aid in recruiting bacteria from the environment (34). (C) Ventral dissection of an adult *E. scolopes*. The accessory nidamental gland (ANG) (white outline) lies posterior to the light organ (LO) and directly anterior to the nidamental gland (NG). Developed oocytes are stored in an area at the posterior end of the mantle (white arrow). During oviposition in *E. scolopes*, the oocytes are thought to pass through the oviducal gland (OG) (gray outline), which coats the oocytes in a first layer of jelly and terminates dorsally to the ANG. Scale bars: A = 2 cm; B = 250 µm; C = 1 cm.

The Hawaiian bobtail squid, *Euprymna scolopes*, is a robust model organism for studying host-microbe interactions (Fig. 1A). Primarily studied for its relationship with the bioluminescent bacterium *Vibrio fischeri*, the squid has a specialized light organ equipped with structural features to recruit and maintain *V. fischeri* for light production (5, 8, 23–25). The spatial location of *V. fischeri* within the organ affects bacterial interactions (26, 27), and the organ has physical features that utilize the light produced by the bacteria (28–30). Female bobtail squid have another symbiotic organ called the accessory nidamental gland (ANG), which is part of the reproductive system (Fig. 1C). In contrast to the binary symbiosis found in the light organ, the ANG houses a diverse yet conserved community of bacteria (31, 32). This consortium resides in a complex network of vascularized, epithelium-lined tubules (32), and community analysis via 16S rRNA sequencing shows the ANG of adult *E. scolopes* is dominated by *Alphaproteobacteria* and *Verrucomicrobia*, with *Gammaproteobacteria* and *Flavobacteriia* present at lesser abundances (31–33). These bacteria are environmentally acquired by the early-stage ANG recruitment tissues that are equipped with many ciliated pores and invaginations (Fig. 1B) (31, 34). Unlike the light organ, which is present as the squid hatches, these nascent ANG tissues appear approximately 4 weeks post-hatching (34). As the ANG matures, the relative composition of the bacterial community shifts; early-stage organs are dominated by *Verrucomicrobia,* whereas mature organs are dominated by *Alphaproteobacteria* (34). Notably, if specific members of the bacterial community are not present in the bobtail squid’s habitat, the ANG will not form, suggesting that microbial cues trigger ANG development (35).

ANGs are found in many cephalopods (36–40), and the organ has long been thought to play a role in egg-laying (36, 38, 41). The ANG lies immediately anterior to the nidamental gland (NG), an organ that produces a gelatinous jelly that coats the squid’s eggs (Fig. 1C) (38, 41). It is now understood that the ANG symbiosis is defensive: the bacteria from the ANG are transferred to the egg jelly coat, where they produce antimicrobial compounds and protect the developing embryos from harmful biofouling by fungi and bacteria (31, 32, 42–45). Although it is known that the ANG bacteria are transferred to the egg jelly coat, the specific mechanism by which they are deposited into the jelly coat are not well described.

Sequence and culture-based techniques have revealed key elements of the ANG symbiosis (31–33, 44). However, the lack of spatial understanding leaves foundational questions unanswered. Previous research observed that *Alphaproteobacteria* and *Verrucomicrobia* populations are taxonomically partitioned in separate tubules (32). However, *Gammaproteobacteria* have not been localized in the ANG, and the abundance of tubules containing each taxon has not been quantified. Furthermore, it is not understood if all tubules exclusively contain one taxon of bacteria nor is it known how specific the taxonomic partitioning is; i.e., are bacterial populations also partitioned at lower taxonomic levels, such as genus? To date, structural understanding of ANG tissues has been limited to superficial observations or sectioned tissue, and the three-dimensional architecture of the complicated organ has remained undescribed. Here, we use advanced imaging techniques to visualize the spatial organization of both a complex host organ and the bacterial community that resides within it. The synthesis of these data sheds light on bacterial dynamics within the ANG and informs how the host uses organ structure and spatial organization to shape a symbiotic community involved in host defense.

## MATERIALS AND METHODS

### Animal care and dissections

Animals from five different cohorts were used in this study, each cohort caught between 2017 and 2023. All squid were caught on the shallow flats of Maunalua Bay, Oahu, HI (21°16'51.4"N 157°43'33.1"W) by dip net. Animals were shipped back to the University of Connecticut and housed individually in aquaria for up to three months before euthanasia. Squid were anesthetized in a solution made of artificial seawater and 2-5% ethanol. Upon anesthetization, mantle lengths were recorded, animals were decapitated, and organs dissected. All animals used in this study, with the exception of the sample imaged in Fig. 1B, were adult (mantle length >18 mm) and had fully developed ANGs. The ANGs were dissected out whole, with a portion of the NG still attached in order to visualize the point at which the two tissues meet. Tissues were immediately transferred to a solution of 4% paraformaldehyde in filter-sterilized squid ringers (FSSR) (32) for overnight fixation at 4°C with light rocking. Fixed tissues were washed with three consecutive rinses of FSSR at room temperature and stored in FSSR at 4°C before use.

### Tissue clearing and whole mount imaging

A modified DEEP-Clear (46) technique allowed for optical clearing and depigmentation of whole ANGs from three animals. Briefly, fixed and washed tissues were submerged in pre-chilled acetone at −20°C for 3 h and washed three times with mPBS (50 mM sodium phosphate buffer, 0.5M NaCl, pH 7.4) at room temperature. Samples were then treated with a solution of 75 µg/mL of proteinase K in mPBS for 12 min at room temperature, followed by two washes with a solution of 2 mg/mL glycine in mPBS at room temperature. After three washes of mPBS, tissues were submerged in DEEP Solution 1.1 [10% vol/vol N,N,N′,N′-tetrakis(2-hydroxyethyl)ethylenediamine, 5% vol/vol Triton X-100, 5% vol/vol urea, ultrapure water, pH 10] overnight at 37°C with gentle rotation in the dark. After three mPBS washes, the samples were stained with nuclear stain TOTO-3 iodide (1 µM), and tubules were visualized with *Lens culinaris* lectin conjugated to rhodamine red (20 µg/mL) (Vector Labs) in mPBS with gentle rotation in the dark overnight. After staining, samples were washed with mPBS three times at room temperature and moved back to DEEP Solution 1.1 overnight at 37°C in the dark. Samples were then washed with mPBS and submerged in a 50/50 mix of mPBS and refractive index matching media, made up of 60% wt/wt OptiPrep solution, 20% wt/wt dimethyl sulfoxide (DMSO), 20% wt/wt mPBS, 20 mM Tris, and 1.12 g/mL 5-(N-2–3-dihydroxypropylacetimido)- 2,4,6-triiodo- N,N'-bis-(2,3-dihydroxypropyl) isophthalamide (Nycodenz) (MP Biochemicals). After an 8 h incubation in the dark at room temperature, samples were transferred to refractive index matching media overnight in the dark at room temperature with gentle shaking. The following day, samples were imaged in the refractive index matching media on a Zeiss Light Sheet 7. Resulting image channels were merged, false-colored, and analyzed in arivis Vision4D software (Zeiss). To represent observations of all experimental tissues, tubules were manually traced optical plane by optical plane in one ANG using arivis.

### Embedding and sectioning

Five additional ANGs representing four squid cohorts were sectioned and used for fluorescence *in situ* hybridization (FISH) and hematoxylin and eosin (H&E) staining. Fixed tissues were dehydrated in a graded ethanol series and semi-cleared with three changes of xylenes before being embedded in paraffin wax. Embedded tissues were serially sectioned into 8–10 µm thick sections on a Shandon Finesse 77510250 microtome (Thermo Scientific).

### Fluorescence *in situ* hybridization

Probes for targeting universal bacteria, as well as *Alphaproteobacteria*, *Gammaproteobacteria*, and *Verrucomicrobia* within the ANG were designed based on previously published literature (32, 47–49). Novel probes for targeting *Leisingera* and *Ruegeria* were designed using 16S sequence data in ARB (50) and confirmed for target specificity with TestProbe (51). Novel probes were also experimentally confirmed for target specificity and optimal formamide concentrations using three cultured representative strains of the respective genus. *Leisingera*-specific probes did not target *Ruegeria* and vice-versa (data not shown). All probes were synthesized by Biomers.net (Ulm, Germany). Probe information can be found in Table S1. Due to its limited abundance in the bacterial community (31) and difficulty implementing into the probe set, *Flavobacteriia* were not analyzed in this study.

Sectioned tissues were deparaffinized in three changes of xylene and dehydrated in three washes of 100% ethanol. FISH probes were applied to sectioned samples at a concentration of 1 µM in a hybridization solution (900 mM NaCl, 20 mM Tris-HCl, 0.02% vol/vol sodium dodecyl sulfate, 20%–30% formamide) without light in a humidifying chamber at 46°C for 3 h. Probes of similar formamide concentrations were hybridized together. If hybridization with another formamide concentration was performed, samples were submerged in wash buffer (112–225 mM NaCl, 20 mM Tris-HCl, 5 mM EDTA, 0.01% vol/vol sodium dodecyl sulfate) at 46°C for 20 min, the other probes and hybridization buffer were applied, and washing was repeated. Slides were counterstained with 5 µg/mL 4’,6-diamidino-2-phenylindole (DAPI) at room temperature for 10 min without light. Slides were then dipped in cold Nanopure water, air dried in the dark, and mounted in Prolong Gold antifade reagent (Invitrogen) with a no. 1.5 coverslip. Mounted slides were cured overnight at room temperature in the dark prior to imaging.

Hybridized slides were imaged on a Nikon A1R laser scanning confocal microscope. For Combinatorial and Spectral Imaging FISH (CLASI-FISH), spectral images were acquired by sequentially illuminating the sample with 640-, 561-, 514-, 488-, and 405-nm laser lines using a 40× Plan-Apochromat 1.3 NA lens or 60× Plan-Apochromat 1.4 NA lens. Linear unmixing was performed with Nikon NIS-Elements software (Nikon) using reference spectra acquired from cultured strains hybridized with the appropriate fluorophores and imaged similarly to samples. The strongest signal for each unmixed spectrum was used for each fluorophore. Unmixed images were assembled and false-colored in Fiji (52). For images with dense bacterial populations, a background subtraction was performed on unmixed images to resolve individual cells. To verify spectrally acquired images, the conjugated fluorophores of the probes were swapped, and hybridization and imaging were repeated to recapitulate similar images (Table S1).

To acquire larger images of whole ANG sections for the quantification of tubules, we used a traditional FISH approach without spectral imaging. These slides only had FISH probes targeting *Alphaproteobacteria*, *Gammaproteobacteria*, and *Verrucomicrobia*, hybridized as described above. Eubacterial universal probes were applied to serial sections to verify bacterial presence in the taxa-specific sections. Hybridized slides were imaged on a Nikon A1R laser scanning confocal microscope using a 20× Plan-Apochromat 0.75 NA lens and illuminated with 405-, 488-, 561-, and 640-nm laser lines. Tubule counts were analyzed and quantified by hand in Fiji (52).

### Hematoxylin and eosin staining of ANG sections

Deparaffinized slides were rehydrated through 5 min washes in a graded ethanol series, followed by 5 min in tap water. Sections were submerged in Gill’s 2 hematoxylin for 3 min before gentle rinsing under tap water for 1 min. Sections were transferred to an acid alcohol differentiation solution for 1 min, rinsed again in tap water for 1 min, and then stained with an Eosin Y solution for 1 min. After a 1 min rinse in tap water, sections were dehydrated again with 2 min washes in a graded ethanol series and two 3 min washes in xylene. Sections were mounted with Permount, allowed to cure overnight and imaged on a Leica Thunder Imager with a 63 × 1.40 Plan Apochromat lens. Resulting RBG images were composited and false-colored in Fiji (52).

### Transmission electron microscopy (TEM)

Squid and ANGs that were collected and processed for TEM in a previous study (32) were used again for this study. Briefly, samples were fixed in 2.0% paraformaldehyde-2.5% glutaraldehyde in buffer A (0.1 M sodium cacodylate, 0.375 M NaCl, 1.5 mM CaCl2, 1.5 mM MgCl2, pH 7.4). Samples were washed in buffer A and then post-fixed in a solution of 1% osmium tetroxide, 0.8% potassium ferricyanide, 0.1 M sodium cacodylate, 0.375 M NaCl for 1.5 h at 4°C. Upon washing and dehydration, samples were embedded in a mixture of Embed 812 (Electron Microscopy Sciences) and Araldite 506 (Ernest Fulham Inc.). Thin sections were obtained using a diamond knife on a LKB Ultramicrotome V and stained with 2% uranyl acetate and Reynold’s lead citrate and viewed with an FEI Tecnai Biotwin G2 Spirit electron microscope operated at 80 kV.

## RESULTS

### Adult ANG structure

**Fig 2:**
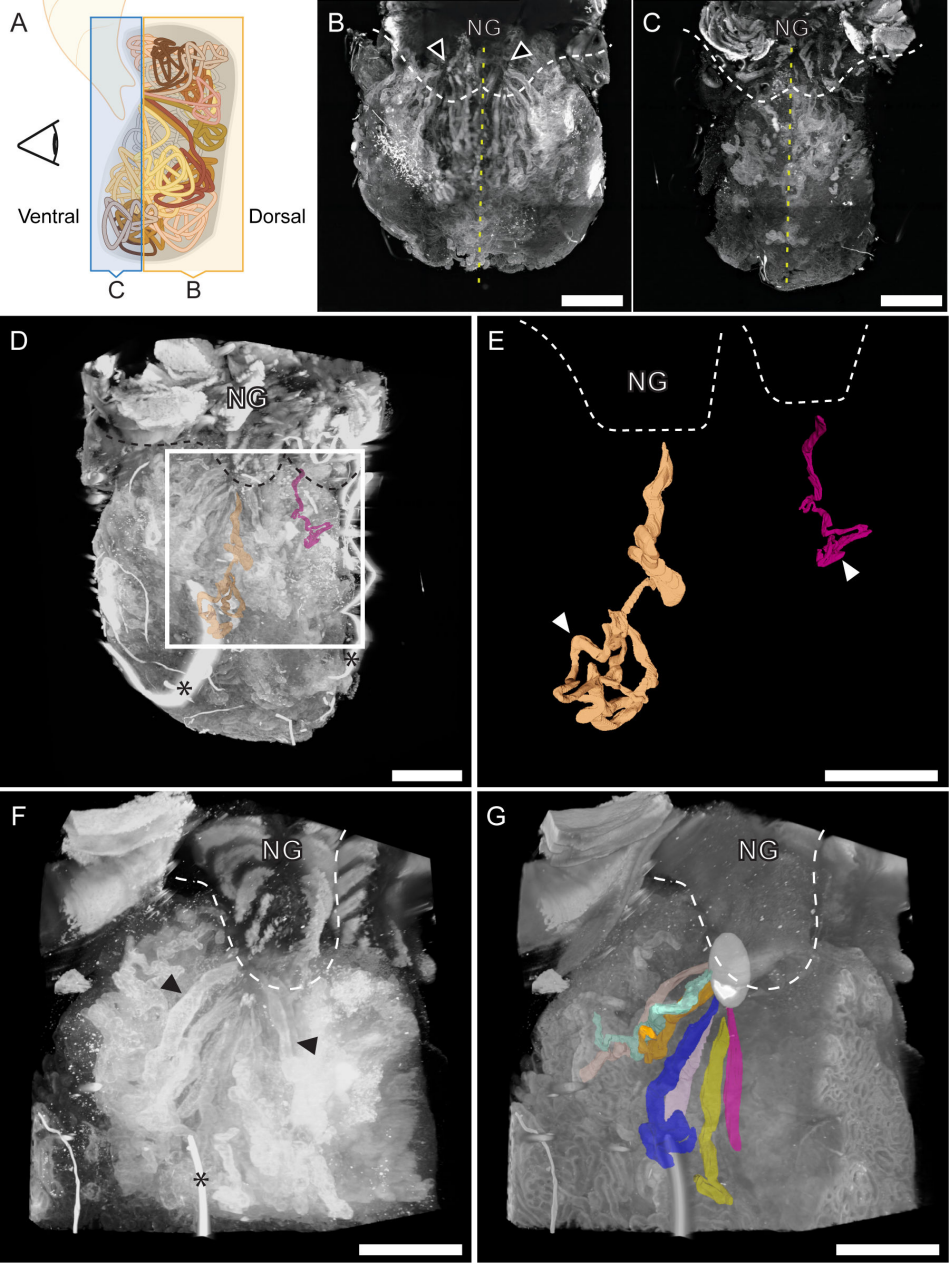
Three-dimensional organization of the ANG. Representative images of cleared whole-mount ANG tissue stained with *Lens culinaris* lectin and TOTO-3 iodide. All images captured on the Zeiss Lightsheet 7 microscope and analyzed in arivis. (A-C) ANG tubules on the ventral side of the organ cross the medial line, making the bi-lobed organ appear as one lobe. (A) Cartoon depicting the optical planes displayed in the maximum intensity projections in panels B and C. (B) Maximum intensity projection of the dorsal 2/3 of the organ. The middle of the organ displays bilateral symmetry across the medial line (yellow dashed line), and the bilobed structure of the organ is apparent. Tubules converge at a single point on each lobe of the organ (black arrowheads). (C) Maximum intensity projection of the ventral one-third of the organ. Superficial, ventral tubules are unorganized and cross the medial line (yellow dashed line), which makes the organ superficially appear as one lobe. (D) Maximum intensity 3D rendering of entire ANG tissue, with representative traced tubules labeled within. (E) Enlarged image of boxed area in D, showing two representative traced tubules. Individual tubules coil in a discrete region (white arrowheads) and lead toward the NG. (F) Maximum intensity 3D rendering of a quadrant of ANG showing individual tubules (black arrowheads). (G) Surface 3D rendering of the same quadrant in F. Representative traced tubules converge together at a point (white ovoid) dorsal and adjacent to the NG. White dashed lines in B, C, E, F, and G and gray dashed lines in D denote the anterior border of the NG. Asterisks in D and F denote dust contaminants. Scale bars: B-E = 1,000 µm; F&G = 500 µm. Video S1 depicts 3D data shown in B-E, and Video S2 shows 3D data in F&G.

A combination of classical and advanced light microscopy methods revealed newly described features and organization of the adult ANG tissue. Optical clearing and fluorescent staining allowed for visualization of the three-dimensional structure of the ANG tubules via light sheet microscopy (Fig. 2; Video S1). In the center of the ANG, large tubules converged toward the nidamental gland (NG) (Fig. 2B through E; Video S1). The tubules converged at two different points, directly underneath each half of the NG (Fig. 2B, F, and G; Video S2). In the middle of the organ, these converging tubules did not cross over the medial line of the ANG, creating the appearance of a two-lobed structure in the middle/dorsal region of the gland (Fig. 2B). As tubules reached the lateral and ventral superficial surface of the gland, the individual clustered tubules did cross the medial line and obfuscate the bilobed structure of the organ. (Fig. 2C). Manually tracing an individual tubule from the convergence point showed that it was non-intersecting and ran continuously before it narrowed and eventually tightly bundled upon itself in a confined space (Fig. 2D and E; Video S1).

**Fig 3:**
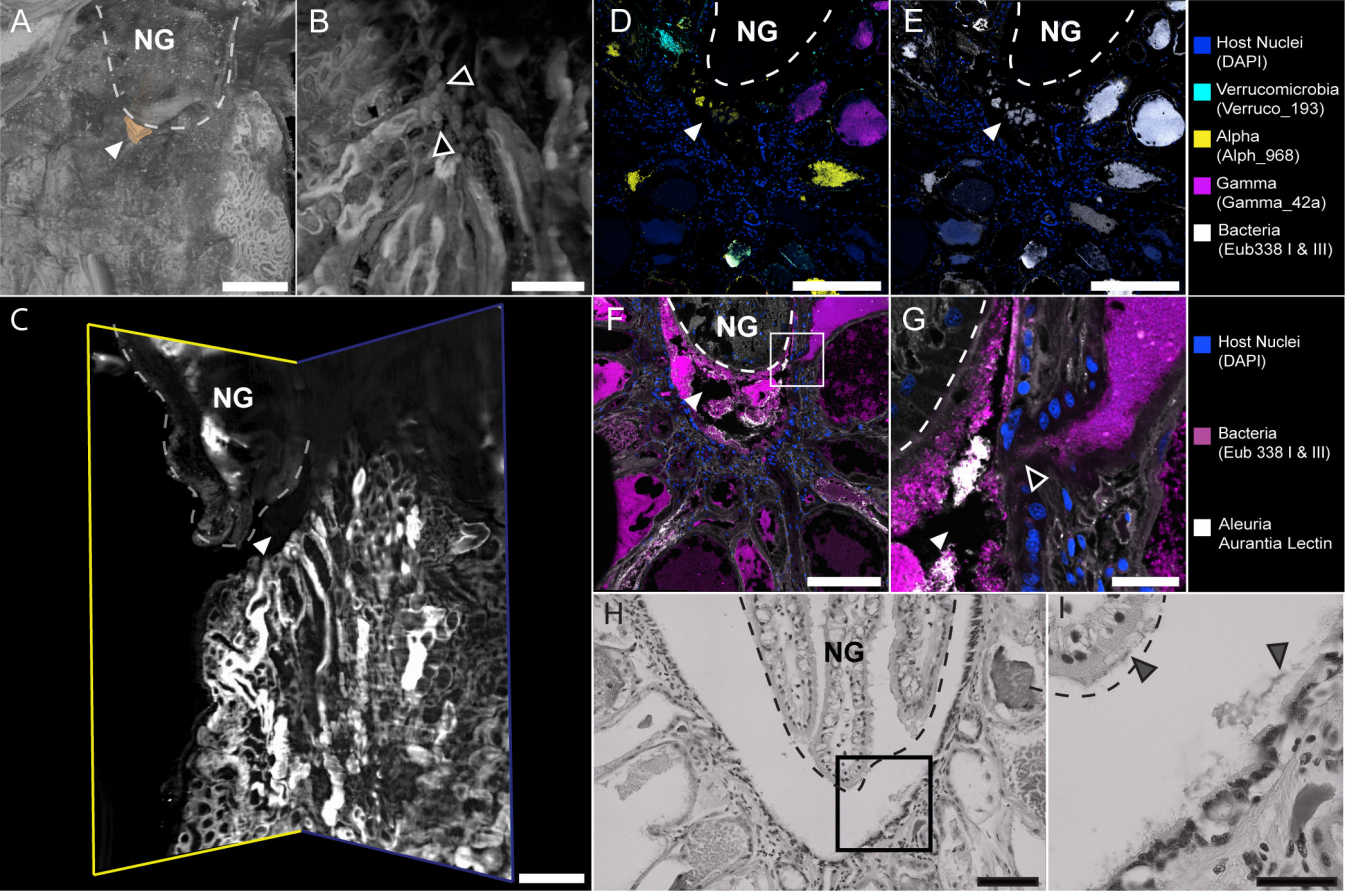
The intergland space. (A-C) Cleared, whole-mount tissue stained with *Lens culinaris* lectin and TOTO-3 Iodide imaged on a Zeiss Lightsheet 7 microscope. (A) Surface 3D rendering of ANG and NG. At the point of tubule convergence, there is an open space between the ANG and NG (white arrowhead/orange region), henceforth referred to as the “intergland space.” (B) ANG tubules terminate at pores that line the intergland space (black arrowheads). (C) Clipped surface 3D rendering of ANG and NG, showing the intergland space (white arrowhead) that opens to outside. (D) Combinatorial labeling and spectral imaging FISH (CLASI-FISH) image of sectioned ANG tissue. The three dominant high-level taxa of the ANG are in the tubules converging at the intergland space (white arrowhead). (E) Same section as seen in panel D, labeled with near-universal bacterial FISH probe cocktail. (F) FISH image showing bacteria in converging ANG tubules and in the intergland space (white arrowhead). (G) Enlarged image of boxed region in F showing that bacteria have access to intergland space (white arrowhead) via tubule pore (black arrowhead). (H) Hematoxylin and eosin (H&E) stained section of intergland space. (I) Enlarged image of boxed region in H. Cilia line the intergland space and contain bacteria-like debris (grey arrowheads). White dashed line in A-G, and gray-dashed line in H&I denotes the anterior boundary of the NG. Scale bars: A = 250 µm; B = 50 µm; C = 200 µm; D-F = 200 µm; G = 20 µm H =100 µm; and I = 50 µm. Video S3 depicts 3D data shown in A–C.

We further investigated the point where ANG tubules converged underneath the NG. On the posterior end of the ANG, a channel in the gland creates a small space between the ANG and NG (Fig. 3A; Video S3). Here, tubules terminate at pores that empty into this “intergland space” (Fig. 3B and C; Video S3). The intergland space of one lobe was lined with approximately 125 pores, suggesting that there are 125 tubules in that lobe of the ANG. With the use of FISH, we observed *Alphaproteobacteria, Verrucomicrobia*, and *Gammaproteobacteria* within these converging tubules (Fig. 3D and E). In freshly dissected animals, we observed a peristaltic motion of ANG tubules that appeared to push the tubule contents toward the intergland space (Video S4). Confocal and lightsheet microscopy showed that these tubules opened into the intergland space (Fig. 3F and G), and bacterial taxa observed in the ANG tubules were also observed in the intergland space (Fig. 3D through G), suggesting that bacteria are secreted from the ANG into the intergland space. H&E staining of tissue sections revealed that cilia line the intergland space on both the NG and ANG. (Fig. 3H and I). These cilia contained bacteria-sized particles (Fig. 3I), which were confirmed to be bacteria using FISH (Fig. 3G).

### Host cells interact with ANG bacteria

**Fig 4:**
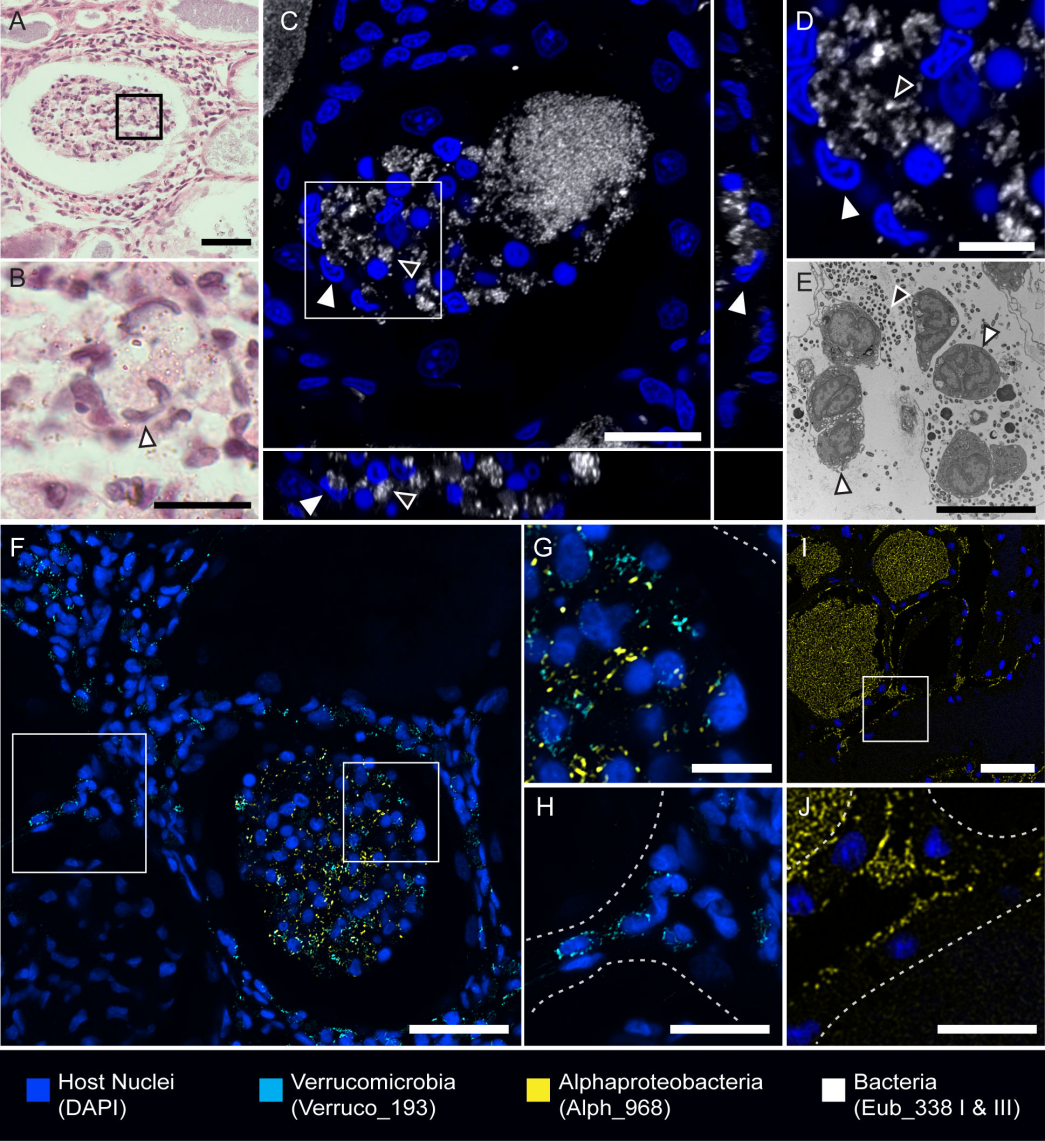
Host cells that resemble hemocytes cluster in ANG tubules, and bacteria are found in interstitial spaces. (A) H&E-stained section. Host cells in eosinophilic material in tubules. (B) Enlarged image of boxed region in A. Host cells resemble hemocytes (white arrowheads) and surround bacteria-like debris. (C) Orthogonal projection of FISH image of sectioned tissue. Hemocyte-like cells (white arrowheads) surround and may engulf bacteria (black arrowheads). (D) Enlarged image of boxed region in C. (Arrowheads indicate the same as in C). (E) Transmission electron micrograph (TEM) of hemocytes (white arrowheads) inside tubules interacting with bacteria (black arrowheads). (F-H) FISH image of sectioned tissue. (G) Enlarged image of boxed region in F of hemocyte-like cluster. Bacteria in the hemocyte-like clusters are of mixed taxa, containing *Alphaproteobacteria* and *Verrucomicrobia*. (H) Enlarged image of boxed region in F, showing *Verrucomicrobia* cells in the interstitial space between tubules. (I) FISH image of sectioned tissue showing *Alphaproteobacteria* residing in tubules and in interstitial space. (J) Enlarged image of boxed region in I, showing *Alphaproteobacteria* in between tubules. White dashed lines in G, H, and J denote tubule borders. Scale bars: A = 100 µm; B = 50 µm; C = 20 µm; D&E = 10 µm; F = 75 µm; G = 10 µm; H = 20 µm; I = 50 µm; and J = 20 µm.

In most tissue sections, we observed clusters of host cells within the tubule lumina, often toward the posterior end of the organ near the tubule convergence point (Fig. 4). These host cell clusters contained bacteria-like debris in an eosinophilic substance, visualized on H&E-stained sections (Fig. 4A and B). The nuclei of host cells in the clusters had the hallmark horseshoe shape of hemocytes, the macrophage-like squid immune cells that circulate in the blood (53–55). FISH imaging showed that the host cells were interacting with and—in some cases—possibly engulfing bacteria within the tubule lumina (Fig. 4C and D). Transmission electron microscopy also showed host cells with a hemocyte morphology interacting with bacteria inside the tubule lumina (Fig. 4E). Imaging with taxa-specific FISH probes revealed that mixtures of both *Alphaproteobacteria* and *Verrucomicrobia* were associated with these host cell clusters (Fig. 4F). *Alphaproteobacteria* and *Verrucomicrobia* were also both found in the interstitial tissue space between tubules (Fig. 4H through J). Bacteria were observed in the interstitial space throughout tissue sections, and the tissues appeared undamaged, suggesting that the presence of bacteria in the interstitial space was not an artifact of tissue processing. *Gammaproteobacteria* were not observed in the interstitial space.

### Distribution of bacterial taxa in the ANG

**Fig 5:**
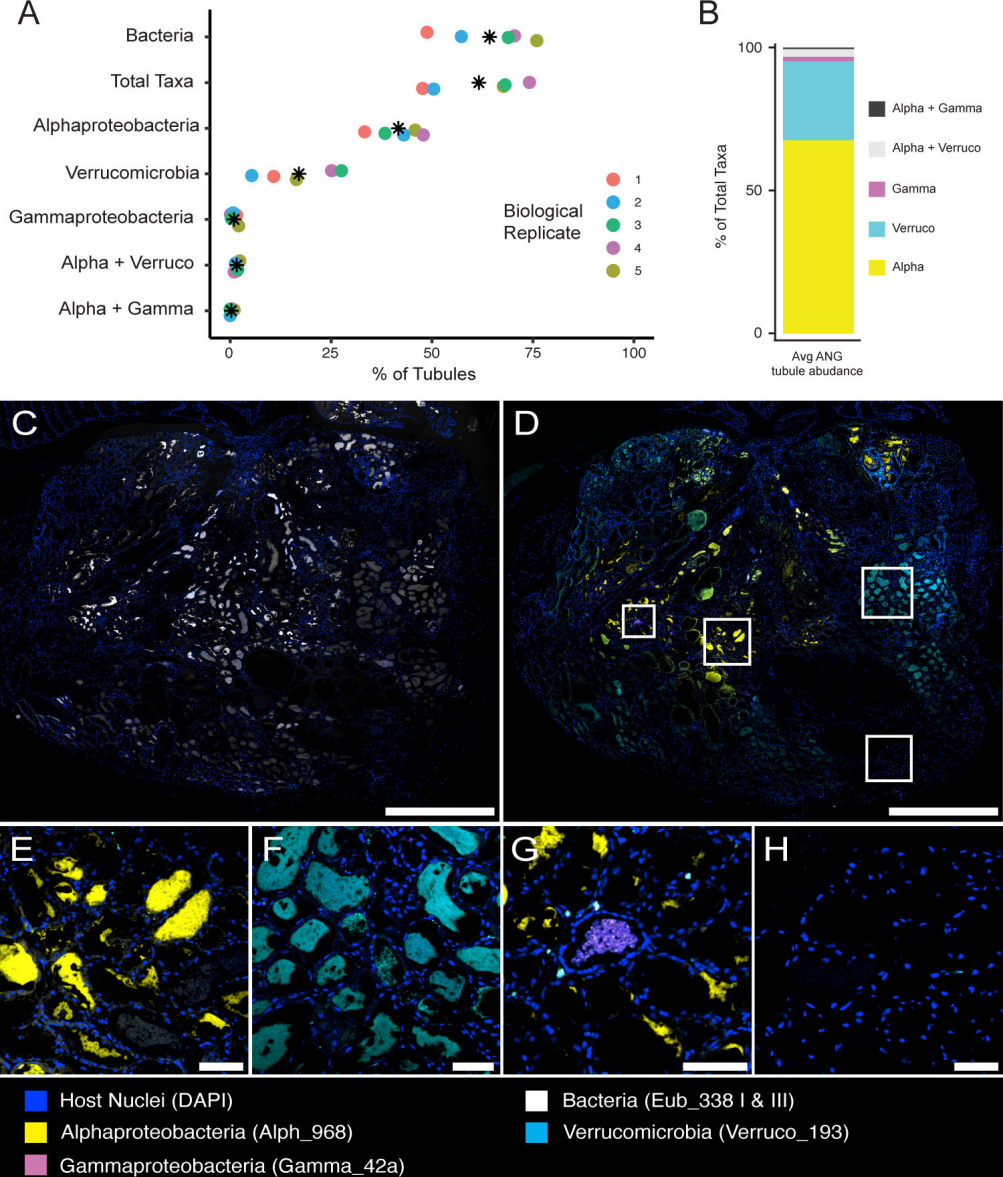
Tubule abundances in the ANG. (A) Abundance of tubules containing each bacterial probe. “Total taxa” is the summed abundance of tubules containing any of the three represented taxa probes. Asterisks denote mean. (B) Relative abundance of the “Total-taxa” tubules that contain each taxon. Five ANGs were used to calculate abundance statistics. (C) FISH image of a section of an entire ANG, labeled with near-universal bacterial FISH probe cocktail used as a positive control. (D) A section serial to panel C, showing the three most abundant higher-level bacterial taxa in the ANG. (E–H) Enlarged images of boxed regions in D, showing tubules dominated by exclusively (E) *Alphaproteobacteria*, (F) *Verrucomicrobia*, (G) *Gammaproteobacteria*, and (H) no bacteria. Scale bars: C&D = 1,000 µm; E–H = 100 µm.

**Fig 6:**
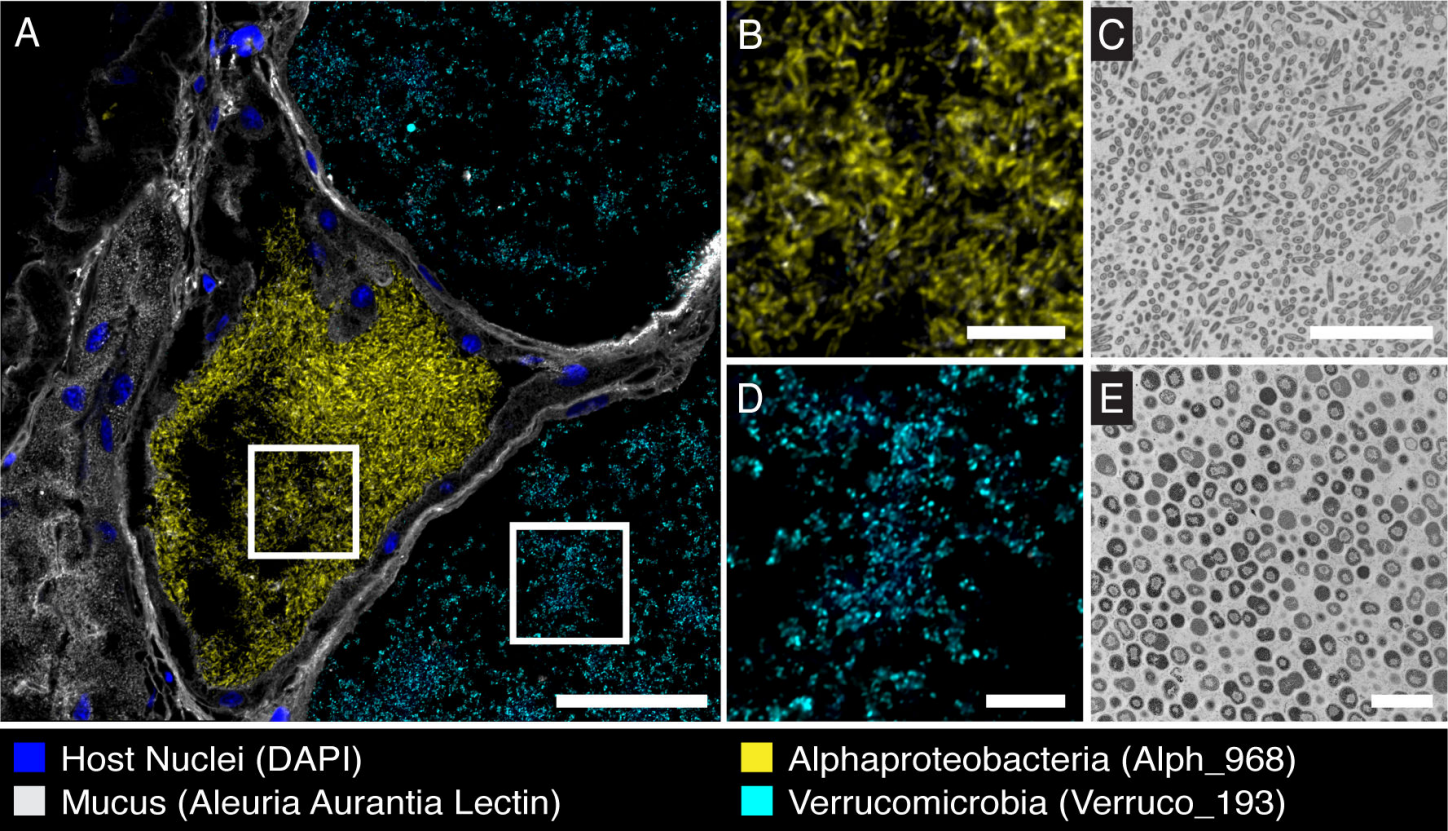
Bacterial taxa correlate with cell morphology. (A) FISH image of sectioned tissue. (B) Enlarged image of boxed region in A. *Alphaproteobacteria* were the only bacteria observed as rods in FISH images. (C) TEM of rod-shaped bacteria in ANG in ANG tubules. (D) Enlarged image of boxed region in A. *Verrucomicrobia* were only observed as cocci. (E) TEM of cocci bacteria in ANG tubules. Scale bars: A = 75 µm; B = 15 µm; C = 10 µm; D = 15 µm; and E = 5 µm.

**Fig 7:**
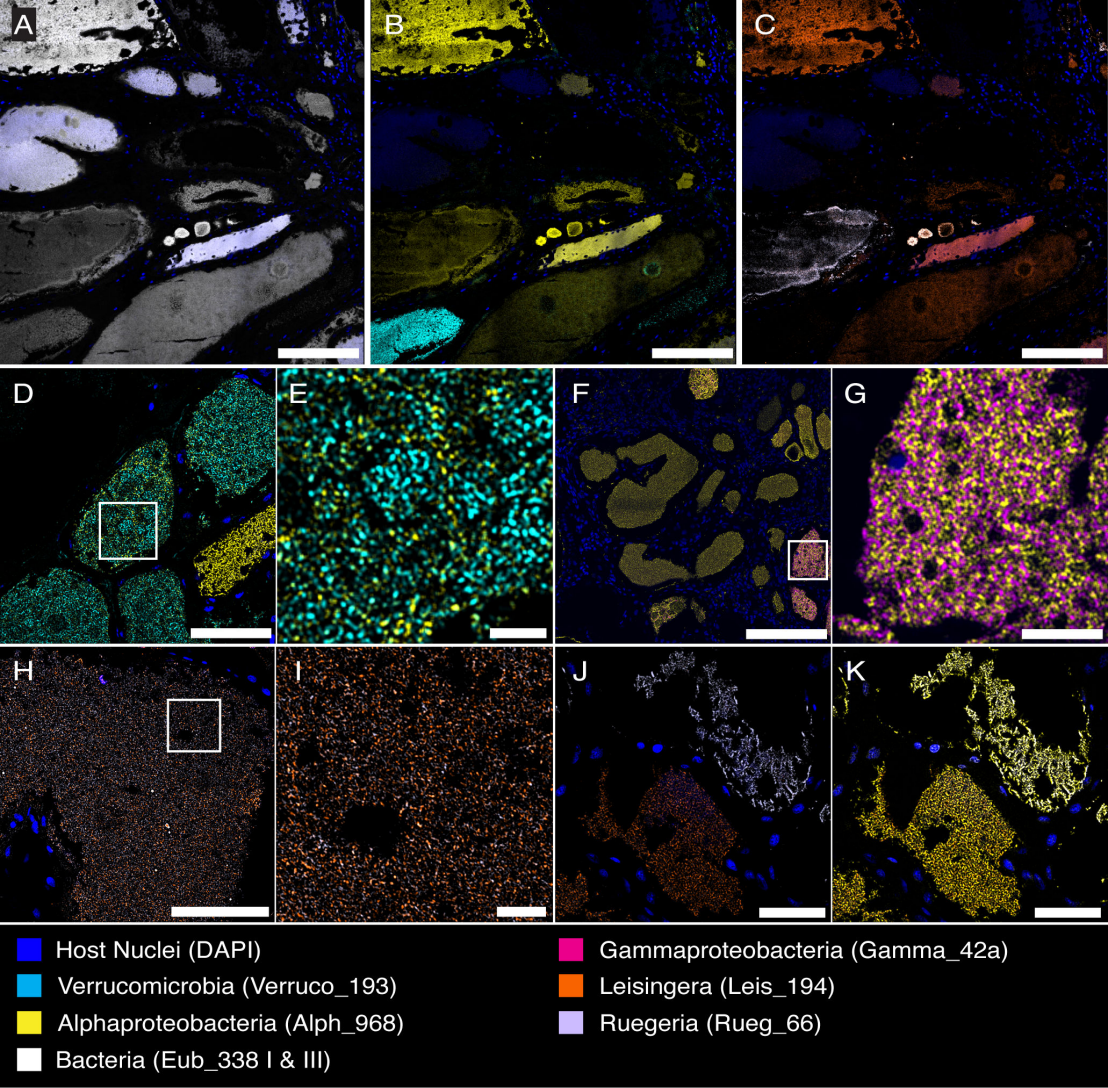
Tubules have mixed and dominant populations of bacteria at different taxonomic levels. CLASI-FISH images of sectioned tissues. (A-C) CLASI-FISH allows for visualization of many bacterial taxa on the same tissue section. Labeling with (A) near-universal bacteria FISH probes, (B) higher-level taxa probes, and (C) genus-level probes can all be done on the same sectioned slide. (D) *Alphaproteobacteria* and *Verrucomicrobia* in the same tubule. (E) Enlarged image of boxed region in D. (F) *Alphaproteobacteria* and *Gammaproteobacteria* in the same tubule. (G) Enlarged image of boxed region in F. (H) The genera *Leisingera* and *Ruegeria* of class *Alphaproteobacteria* were found in the same tubule. (I) Enlarged image of boxed region in H. (J) These genera were also seen dominating unique tubules. (K) *Alphaproteobacteria* probe overlay of J. Scale bars: A-C = 200 µm; D = 50 µm; E = 10 µm; F = 200 µm; G = 20 µm; H = 50 µm; I = 5 µm; and J&K = 50 µm.

Hybridization with the near-universal Eub338I and III probe cocktail showed that a majority of tubules (64.2% ± 9.9%) contained detectable bacteria (Fig. 5A through C). The communities of bacteria within tubule lumina were often densely populated and displayed different organizations between different tubules. Some tubules were full of bacteria, whereas others contained different bacterial arrangements (Fig. 5 to 7). These morphological differences were independent of which bacterial taxa resided within the tubules and were observed regardless of fixation method (Carnoy’s-fixed images not shown) and in whole-mount tissues, suggesting that these different arrangements were not a product of tissue sectioning.

In the five ANGs surveyed, we observed *Alphaproteobacteria* in all tissue sections and in the greatest number of tubules (41.7% ± 5.3%) (Fig. 5). Tubules containing *Alphaproteobacteria* were most commonly found in the center of the organ and in the tubules converging underneath the NG. These *Alphaproteobacteria* were densely packed, at times making it difficult to perceive individual cells and bacterial morphology. Members of *Alphaproteobacteria* were the only ANG bacteria in which both cocci and rod bacterial morphologies were observed (Fig. 4G, 6B and C). *Alphaproteobacteria* were the most dominant members of the bacterial community observed in the intergland space between the ANG and NG (Fig. 3D).

*Verrucomicrobia* were observed in the second greatest number of tubules (17.1% ± 8.4%) and found in all tissue sections (Fig. 5). Although tubules containing *Verrucomicrobia* were located near the convergence point, they were commonly found around the periphery of the organ and contained both loosely and densely packed populations. *Verrucomicrobia* populations were only observed to be of cocci morphology (Fig. 6D and E). Tubules containing *Gammaproteobacteria* were the least abundant (0.96% ± 0.77%) and were densely packed (Fig. 5).

Generally, similar bacteria were seen in tubules clustered together (Fig. 5), likely due to a tubule passing through the sectional plane multiple times as it clusters in a discrete region of the ANG (Fig. 2E). Some tubules were labeled with neither the Eub338 cocktail FISH probes nor the taxa-specific FISH probes. Tubules lacking detectable probe signals were often large, and although they were at times found at the convergence point of tubules, they were most often found distal to the convergence point near the intergland space (Fig. 5H).

CLASI-FISH imaging allowed us to observe multiple bacterial taxa within the same tissue section (Fig. 7A through C). Although most tubules were dominated by a single taxon (Fig. 5), we also observed tubules with mixed populations of bacterial taxa (Fig. 7). These mixed tubules contained populations of *Alphaproteobacteria* and *Gammaproteobacteria* or *Alphaproteobacteria* and *Verrucomicrobia* (Fig. 5B). These tubules were found near the center of the organ and adjacent to identically populated tubules or those dominated by exclusively *Alphaproteobacteria*. The distribution of bacteria in mixed populations varied; bacterial taxa were both homogeneously (Fig. 7F and G) and heterogeneously (Fig. 7D and E; Fig. S3) distributed in the lumina, regardless of which taxa were present. Tubules of mixed taxa always contained *Alphaproteobacteria*, and tubules containing *Gammaproteobacteria* and *Verrucomicrobia* were not detected. Although tubules containing mixed populations were infrequent, they were observed in every biological replicate (*n* = 5).

Interestingly, similar observations were made with FISH probes targeting bacterial genera. In the ANG, the two most populous genera within the class *Alphaproteobacteria* are *Ruegeria* and *Leisingera* (31). These two groups were observed both individually dominating some tubules (Fig. 7J) and in mixed populations together (Fig. 7H and I). Not all tubules containing *Alphaproteobacteria* probes were labeled with these genus-specific probes, suggesting that other *Alphaproteobacteria* genera reside in these tubules.

## DISCUSSION

Host-associated microbiome studies often focus on microbial community diversity but lack spatial context. By imaging the symbiotic bacterial community of the *E. scolopes* ANG in relation to the host, we gain a better understanding of how the host houses and uses its symbiotic microbial community. To date, structural analysis of the cephalopod ANG has been limited to two-dimensional tissue sections. By pairing these classical methods with advanced light microscopy, we resolve the complex host organ structure and the spatial organization of the bacterial symbionts.

### The ANG structure informs defensive function

**Fig 8:**
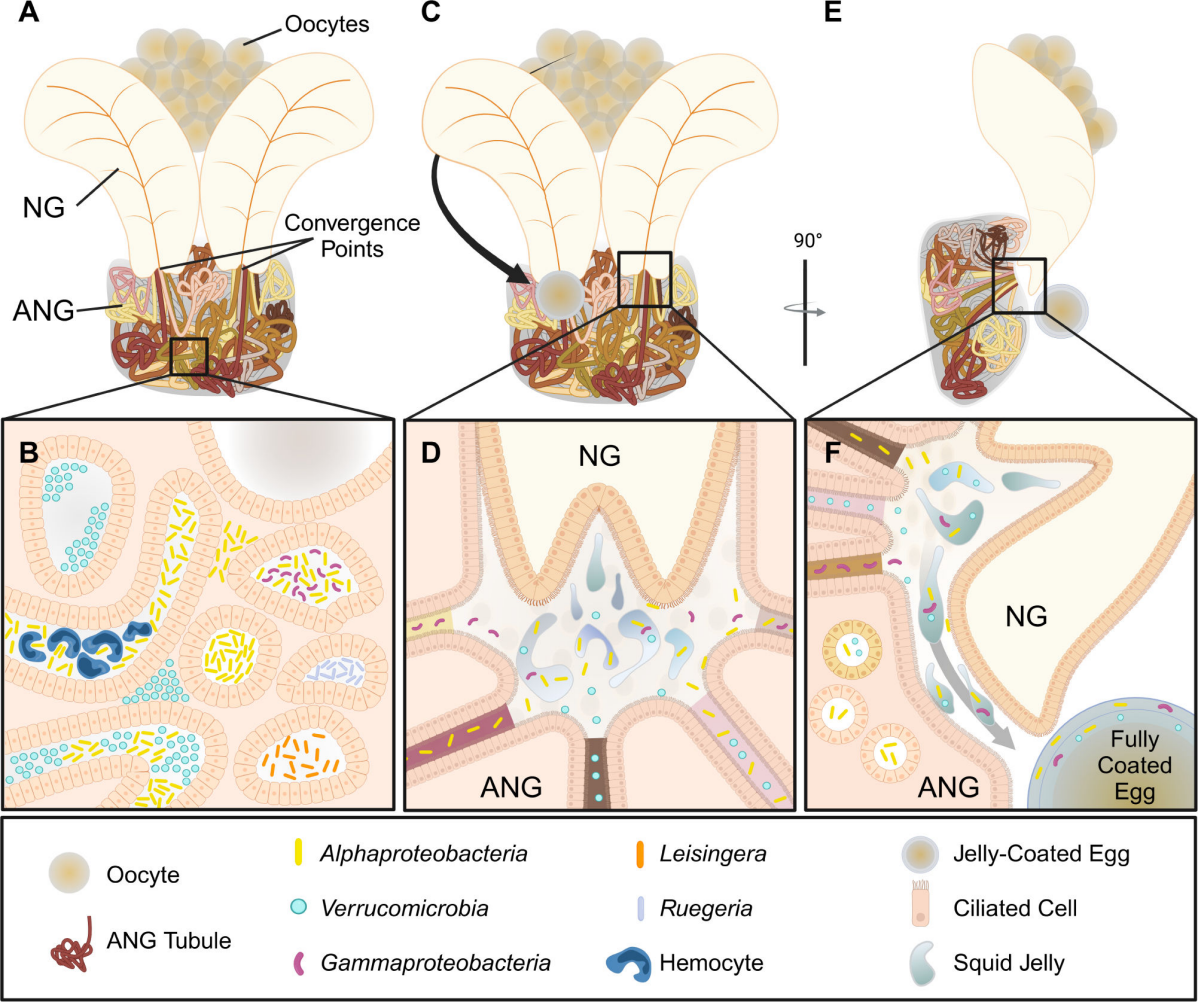
Model of ANG structure and symbiont microbiogeography during egg deposition. (A-B) Representative ANG structure and bacterial organization. (A) Individual tubules bundle together in a defined region of the organ. Tubules converge underneath each lobe of the nidamental gland (NG). Unprocessed oocytes are found in ovaries posterior to the NG. (B) The orientation of bacterial taxa and structural arrangements observed in the ANG tubules. (C-F) Proposed mechanism of bacterial deposition on eggs. (C) The oocyte travels near the opening of the intergland space, where ANG tubules converge and open. (D) The intergland space. Tubules converge toward, open to, and deposit bacteria in the intergland space. Jelly from the NG is expelled into intergland space, and ciliary action mixes the bacteria and jelly to coat the oocyte with antimicrobial-producing symbionts. (E) Rotation of organ to highlight the direction of jelly and bacteria deposition during egg laying. Converging tubules open into the intergland space. (F) Cilia transport the bacteria-jelly mixture out of the intergland space onto the now fully coated squid egg. Created in BioRender [Kamp, D. (2025) https://BioRender.com/j47g955].

The complex 3D organization of tubules visualized with light sheet microscopy illuminates how bacteria are stored in the ANG and deposited onto eggs. No tubules were observed intersecting with each other, neither in whole-mount organs nor sectioned tissues. Thus, it appears that the ANG is a composite organ of many individual tubules, each containing its own bacterial population. The organ appears divided into two lobes, each terminating underneath the NG (Fig. 8A and E). This bilobed structure is superficially evident in dissections of other cephalopods; in cuttlefish, there are two distinct hemispheres of the ANG (37, 39, 41). To our knowledge, this is the first description of tubule convergence in a cephalopod ANG. The crowding of tubules on the ventral surface of the organ previously masked this division and convergence in the adult gland of *E. scolopes*. The medial division of the adult gland mirrors the bilobed, ciliated epithelial fields observed earlier in development of the *E. scolopes* ANG (34) and suggests that the two lobes never truly merge. Rather, the tight packing of superficial tubules forms the appearance of a single-lobed organ.

The spatial layout of ANG tubules and presence of bacteria in the intergland space reveal how bacteria may be deposited in the egg jelly coat. During oviposition, an oocyte is transported in front of the intergland space, likely via the oviduct and oviducal gland, as described in other cephalopods (Fig. 8C) (41). The intergland space serves as the location where the symbiotic bacteria from the ANG mix with the jelly produced by the NG before encapsulating the oocyte and forming the coated egg (Fig. 8D). The peristaltic motion of ANG tubules pushes the tubule’s bacterial contents into the intergland space (Video S4), and the cilia lining the NG and ANG in the intergland space may facilitate the mixing of bacteria and jelly as well as the transport of the mixture to the egg. This mixing of bacteria and jelly during egg encapsulation allows the bacteria to be evenly deposited in the jelly coat of the egg (Fig. 8F). Other microscopic studies of ANGs observed tubules terminating and emptying at the outer surface of the ANG (38) or directly penetrating and emptying bacterial contents into the NG (39), but an intergland space has not been previously described. The three-dimensional observations of this study, together with classical tissue sectioning, suggest a mechanism by which the host bobtail squid distributes symbiotic bacteria in jelly coats of eggs during oviposition to assist in egg defense.

Symbioses involved in egg defense are prevalent in nature, and both aquatic and terrestrial animals have evolved mechanisms to employ symbiotic bacteria (56). Epibiotic bacteria associated with a variety of crustacean eggs have been shown to protect the eggs from fungal fouling (57–59). Although some of these studies were able to identify the specific microbes and compounds involved in the defenses (58, 59), how the host selects, maintains, and deposits these symbiotic bacteria on the eggs remains unknown. Some defensive symbioses in the terrestrial environment also lack a directly observed host source for symbiont deposition: eggs of the non-biting midges *Chironomidae* contain *Firmicutes* bacteria that potentially protect the eggs from heavy metals (60), and eggs from the housefly *Musca domestica* contain bacteria that prevent fungal fouling (61). However, organs transferring defensive symbionts have also been identified in some terrestrial insects. Female beewolves, *Philanthus triangulum*, coat developing larvae with *Streptomyces* bacteria that protect the developing larva via the production of antimicrobials (62–64). These symbionts are housed in specialized antennal glands and are applied to the brood pouch, where they are then transferred to the larval cocoon. The beetle *Lagria villosa* has tubular accessory glands that harbor *Burkholderia* symbionts that also protect eggs via antimicrobial production (65–67). Notably, the symbionts of these insects are vertically transmitted from mother to offspring. In contrast, the ANG microbial community is horizontally transmitted; each generation of squid acquires a new symbiotic consortium (31). Results from this study show the physical structure of the ANG likely facilitates the long-term maintenance of the symbiotic microbial community, as well as the deposition of these symbionts into the eggs during oviposition.

### Bacterial localization expands understanding of host-microbe interactions in the ANG

Visualization of the ANG bacterial community in this study supports and expands upon prior observations of symbiont localization in the ANG of *E. scolopes*. Both cocci and rod-shaped bacterial cells have been observed in ANG tubules via TEM (32). Using FISH, we determined that both *Verrucomicrobia* and *Alphaproteobacteria* correspond with a coccus cell morphology, but only the *Alphaproteobacteria* were rod-shaped. The same study, using both light and electron microscopy, observed bacteria in the interstitial space between intact tubules (32), and we confirmed that both *Alphaproteobacteria* and *Verrucomicrobia* localized within the interstitial spaces of the ANG. Both *Alphaproteobacteria* (68–71) and *Verrucomicrobia* (72–74) have been observed in intracellular spaces of other hosts ranging from protists to ticks, and future work may determine if these ANG bacteria migrate from the extracellular spaces of the tubule lumina to interstitial or even intracellular spaces. We also observed tubules with varying abundances and arrangements of bacteria, similar to what has been observed in other cephalopod ANGs (36, 38, 39, 75). The movement of bacteria from the tubules into the intergland spaces empties tubules and likely accounts for why some tubules appear devoid of symbionts at any given time. Although our study did not account for when the host last laid eggs, previous observations noted that in other squid, ANG tubules were emptied during spawning (38). Tubules that remain depleted, even distal to the intergland space, may have contained bacteria that have yet to repopulate the lumina, such as the putatively slow-growing *Verrucomicrobia* (76).

The relative abundance of tubules containing each taxon of bacteria resembled the relative abundance of each taxon as estimated via 16S community analysis (31), suggesting that spatial structuring governs bacterial abundance in the organ: the more tubules containing a given bacterial taxon, the more that taxon will be represented in the ANG community. Previous work showed the complete partitioning of bacterial taxa into separate tubules (32), and similar observations were made in this study (Fig. 5E through G). Spatially partitioning specific symbionts is a practice used by many hosts (17), as observed in the light organ of the bobtail squid (8, 25), bacteriocytes of many insects (77), and root nodules of legumes (78). However, the ANG system is, to our knowledge, the only symbiosis that employs such strict physical compartmentalization to sequester and house multiple taxonomically diverse bacterial partners in a single symbiotic organ. This physical partitioning of bacterial taxa may maintain the diversity of antimicrobial-producing bacteria. The many bacterial partners housed in the ANG produce a broad array of antimicrobial products that negatively affect other microbes (4, 42, 43, 45). Experiments show that spatial segregation of bacteria increases the biodiversity in mock communities dominated by negative interactions (79), and host-driven physical structuring of microbial communities on the human skin allows dissimilar strains of bacteria to coexist in the same organ by reducing competition (16). By compartmentalizing bacterial populations, the ANG may reduce within-host conflict. This conflict reduction may benefit microbial taxa that are rare in the host’s environment, such as the *Verrucomicrobia,* which are of low relative abundance in the bobtail squid’s habitat (0.3%) but are one of the dominant taxa in the ANG (25%) (31). Many defensive symbioses involve a limited diversity of bacterial members (56, 62, 66, 67). In the ANG, spatial partitioning may reduce both negative interactions and competition between bacterial community members, thus allowing for a diverse consortium of antimicrobial-producing bacteria that protect the squid eggs from a myriad of fouling pathogens.

A variety of mechanisms may work in concert to maintain a spatially partitioned bacterial population. The conserved bacterial communities between individual ANGs suggest that the host actively selects for specific bacteria from the environment. We observed macrophage-like hemocytes interacting with bacteria in ANG tubules, similar to a previous study (32). Hemocytes are also known to migrate to the light organ, where they interact with *V. fischeri* and play multiple roles in the light organ symbiosis (54, 80–83). In the ANG, they may perform similar functions, including restructuring tissues, regulating symbionts via binding and phagocytosis, or delivering nutrients to the symbionts. Notably, the hemocyte aggregations with eosinophilic material surrounding bacteria in the ANG resemble hemocytes that respond to parasites and pathogens in other invertebrates (84–86), suggesting that the host’s cellular innate immune system may help regulate the consortium, possibly by removing unwanted bacteria from tubules. Furthermore, individual tubules may foster microenvironments that lend a competitive advantage to specific target bacterial taxa, similar to how different bean bugs select for their respective *Burkholderia* symbionts (87). Bacterial interactions may also lead specific taxa to dominate an individual tubule. Bacteria in the *E. scolopes* ANG produce antimicrobial compounds (42, 43, 45), and extracts from other cephalopod ANGs have demonstrated antibacterial activity (75, 88). Further testing may reveal if bacteria producing these antimicrobial compounds dominate individual tubules by eliminating other members via antimicrobial production. Many ANG bacteria also possess type VI secretion systems (44, 89) and may use them to eliminate bacterial competitors in tubules, just as some *V. fischeri* strains do in the light organ crypts (90). These potential inhibitory interactions emphasize the need for the host to partition the bacterial community. Experiments focused on coculturing ANG bacteria with other bacterial strains or the isolatable squid hemocytes (55) will potentially reveal the mechanisms underlying these interactions.

The use of more FISH probes on entire tissue sections revealed that some tubules contain more than one bacterial taxon (Fig. 7). The bacterial interactions in these mixed-population tubules could be syntrophic, such as those observed with mucus-degrading bacteria in the human and insect guts (91–93). However, these inter-bacterial relations may also be antagonistic. The microbial community of the ANG has more *Verrucomicrobia* at the early stages of development but is later dominated by *Alphaproteobacteria* (34). These mixed tubules may result from direct interactions between community members, for example, *Alphaproteobacteria* invading tubules of other bacteria, perhaps by migrating through the interstitial region between tubules or from the intergland space. This spatial localization has physiological implications: the high density at which *Alphaproteobacteria* bacteria inhabit tubules, along with their ability to cohabit with or invade tubules of different bacteria, likely accounts for the group’s predominance in the adult ANG bacterial community.

Understanding how some tubules contain individual genera, whereas others contain mixed phyla may also require further study of the early stage recruitment tissues. The nascent ANG has many small pores primed to recruit environmental bacteria (34), but the mechanisms by which the host selects and separates its specific bacterial consortium are currently unknown. One hypothesis is that stochastic mechanisms, such as ecological drift and the bottlenecking of bacterial populations, drive the partitioning of bacterial consortia. In the human skin, many small pores fragment populations of *Cutibacterium* via neutral bottlenecking (16), and in the squash bug, *Anasa tristis*, symbionts are randomly compartmentalized into different symbiotic crypts (94). Partitioning in the ANG may be driven by similar stochastic effects during colonization. The approximately 700 small pores of the ANG recruitment tissue may be individual bottlenecks that permit very few bacterial cells to enter any given pore (34). These cells could be bacteria from the same taxon, resulting in a tubule dominated by a single kind of bacteria. However, these symbionts may also be of different taxa, whether that be related genera, as observed with the *Ruegeria* and *Leisingera* mixed tubules, or distantly related taxa, as seen with the *Alphaproteobacteria* and *Verrucomicrobia* mixed tubules. Competition between different bacterial groups and/or host factors such as innate immunity or different nutrient microenvironments may then selectively drive the expansion of these initial bacterial inhabitants and maintain partitioning in developing tubules. The combination of the host-derived selection and bacteria-bacteria inhibitory interactions may result in the relative infrequency of tubules with mixed bacterial populations. Experiments in both the bobtail squid light organ and ANG show that the host responds to the presence of specific environmental bacteria (8, 35, 83, 95). Thus, it may be that the initial bacterial community partitioning occurs in the recruitment tissue pores, but the host’s selective maintenance is tailored in response to the specific bacterial population within each developing tubule. Further imaging of the nascent ANG will reveal if and how these spatial divisions structure the adult ANG symbiotic community.

By visualizing organ structure and microbiogeography of bacterial symbionts in the *E. scolopes* ANG, this study revealed how the host fosters and regulates its symbiont community. Three-dimensional imaging clarified the organization of the ANG tubules and revealed direct mechanisms used by the host to deposit the bacteria into the eggs where the bacteria provide antimicrobial protection. In all, the bobtail squid ANG represents a composite system consisting of individual tubules harboring distinct bacterial populations, some of which are dominated by one bacterial taxon while a few contain a mixture of different taxa. We propose that partitioning the bacterial population allows the host to foster a greater diversity of antimicrobial-producing bacteria. Using multidimensional imaging methods, we show that bacterial populations are localized and mappable in a symbiotic organ, and future research correlating bacterial taxa with respective host microenvironments may reveal the cellular and biochemical mechanisms used to select and maintain specific bacteria. The foundation of this study, along with the ability to experimentally introduce bacteria into the developing organ (35), supports the ANG symbiosis in *E. scolopes* as a robust model for understanding the regulation of bacterial consortia in defensive symbioses.

## SUPPLEMENTAL MATERIAL

Supplemental material aem.02163-24-s0001.pdf Table S1, Figures S1 to S3, and legends for Videos S1 to S4.

Video S1 aem.02163-24-s0002.mp4 Three-dimensional features of whole ANG.

Video S2 aem.02163-24-s0003.mp4 Tubule convergence underneath NG.

Video S3 aem.02163-24-s0004.mp4 The inter-gland space in 3D.

Video S4 aem.02163-24-s0005.mp4 Visualizing bacterial deposition from ANG.

ASM does not own the copyrights to Supplemental Material that may be linked to, or accessed through, an article. The authors have granted ASM a non-exclusive, world-wide license to publish the Supplemental Material files. Please contact the corresponding author directly for reuse.
