# Supplementary material for: Organ structure and bacterial microbiogeography in a reproductive organ of the Hawaiian bobtail squid reveal dimensions of a defensive symbiosis
Source: Appl Environ Microbiol. 2025 Apr 15;91(5):e02163-24. doi: 10.1128/aem.02163-24 (PMC12093969; doi:10.1128/aem.02163-24)
Supplement: Supplemental material — Table S1, Figures S1 to S3, and legends for Videos S1 to S4. [file aem.02163-24-s0001.pdf]

Supplemental Figures

| Probe | Target organism | Sequence | Hybridization Buffer % Formamide | Reference | Fluorophores: CLASI-FISH Set 1 | Fluorophores: CLASI-FISH Set 2 | Fluorophores: FISH |
|---|---|---|---|---|---|---|---|
| Eub_338 I | Eubacterial | gctgcctcccgtaggagt | 20 | Amann et. al 1990 | Cy5 | Cy5 | Cy5* |
| Eub_338 III | Eubacterial | gctgccacccgtaggtgt | 20 | Amann et. al 1990 | Cy5 | Cy5 | Cy5* |
| Alph_968 | Alphaproteobacteria | ggtaaggttctgcgcgtt | 20 | Glöckner et. al 1998 | Atto 532 | Dy-610 | Cy3 |
| Verruco_193 | Verrucomicrobia | cgccattacaagctttagta | 20 | Collins et. al 2012 | Texas Red - X | Atto-532 | Cy5 |
| Gamma_42a | Gammaproteobacteria | gccttcccacatcgttt | 20 | Manz et. al 1992 | DY-610 | Texas Red-X | Atto-488 |
| Leis_194 | Leisingera | accgactagcatgctagccg | 30 | This study | Rhodamine Red-X | DY-490 | * Applied to seriel section |
| Rueg_66 | Ruegeria | gctcaccccgaaaggcgcgc | 30 | This study | DY-490 | Rhodamine Red-X | |

**Supplemental Table 1: FISH probes and experimental designs used in study.**

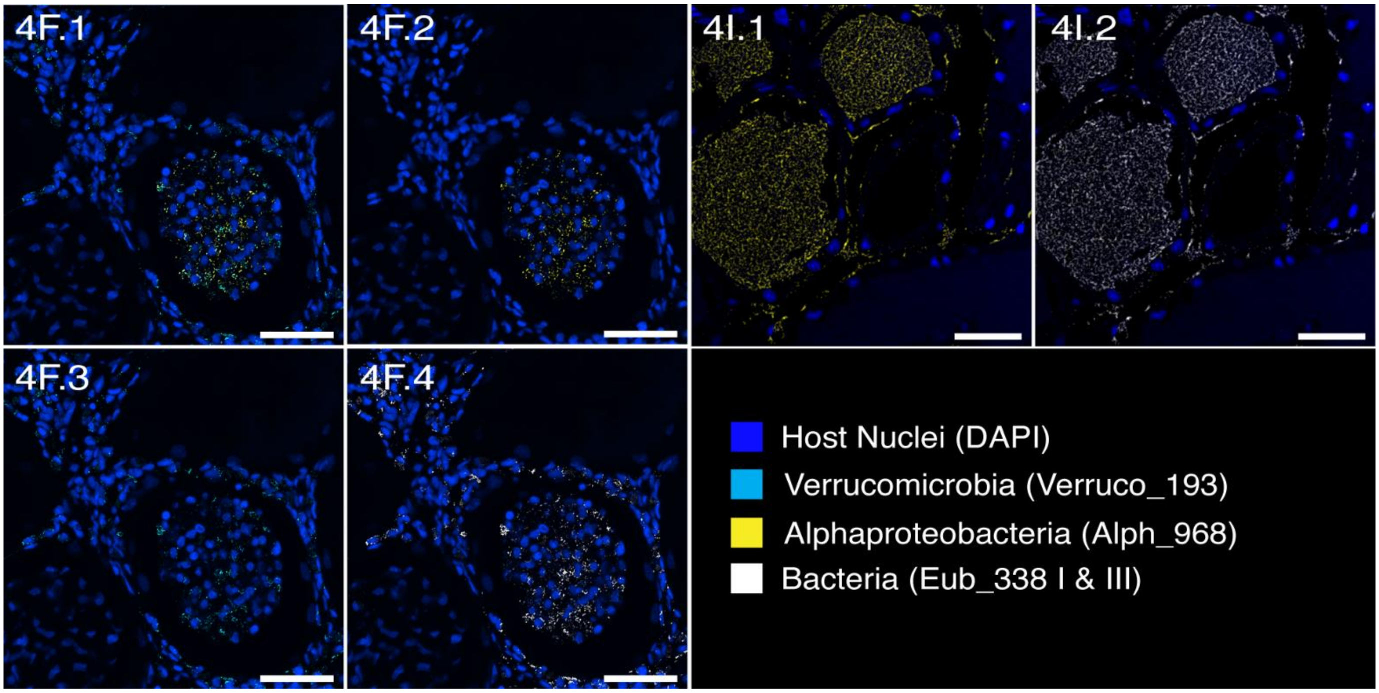


**Supplemental Figure 1: Split image channels and eubacteria probe positive controls for FISH images in Fig. 4.** (4F.1) Image in Fig. 4F. (4F.2) DAPI and Alphaproteobacteria channels. (4F.3) DAPI and Verrucomicrobia channels. (4F.4) DAPI and eubacteria channels. Eubacteria channel shows bacterial distribution nearly identical to that in 4F.1 (4I.1) Image in Fig. 4I. (4I.2) DAPI and eubacteria channels. Bacterial distribution in the eubacteria channel is nearly identical to that in 4I.1 Scale bars: 4F.1-4 = 75 μm; 4I.1&2 = 50 μm

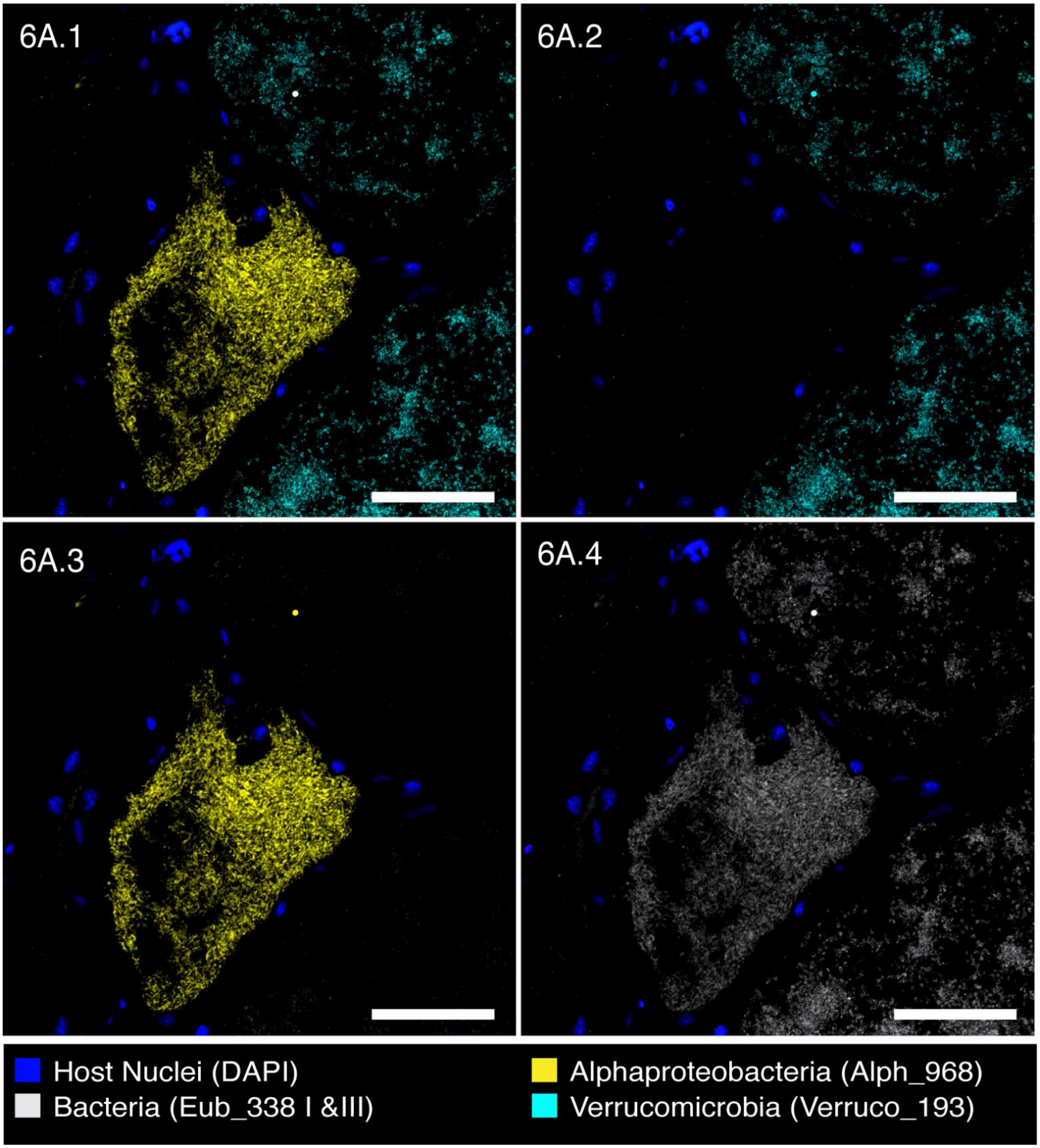


**Supplemental Figure 2: Split image channels and eubacteria probe positive controls for FISH images in Fig. 6.** (6A.1) Image in Fig. 6A. (6A.2) DAPI and Verrucomicrobia channels. (6A.3) DAPI and Alphaproteobacteria channels. (6A.4) DAPI and eubacteria channels. Eubacteria channel shows bacterial distribution nearly identical to that in 6A.1 Scale bars: 75 µm

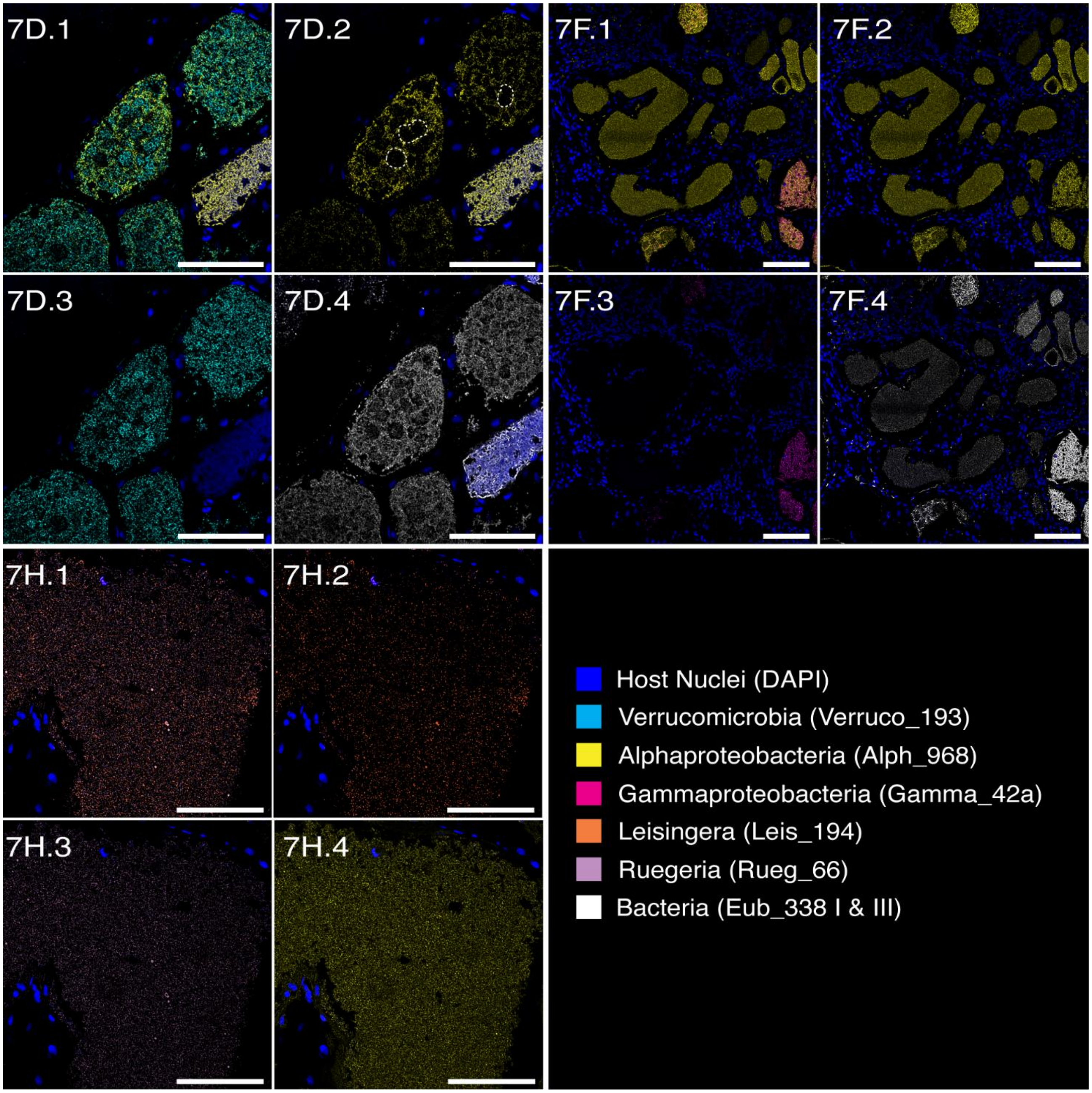


**Supplemental Figure 3: Split image channels and higher taxonomic level probe positive controls for FISH images in Fig. 7.** (7D.1) Image in Fig. 7D. (6D.2) DAPI and Alphaproteobacteria channels. (7D.3) DAPI and Verrucomicrobia channels. (7D.4) DAPI and eubacteria channels. Eubacteria channel shows bacterial distribution nearly identical to that in 7D.1 (7F.1) Image in Fig. 7F. (7F.2) DAPI and Alphaproteobacteria channels. (7F.3) DAPI and Gammaproteobacteria channels. (7F.4) DAPI and eubacteria channels. Bacterial distribution in the eubacteria channel is nearly identical to that in 7F.1 (7H.1) Image in Fig. 7H. (7H.2) DAPI and Leisingera channels. (7H.3) DAPI and Ruegeria channels. (7H.4) DAPI and Alphaproteobacteria channels. Alphaproteobacteria channel shows bacterial distribution nearly identical to that in 7H.1 Scale bars: 7D.1-4 = 50 μm; 7F.1-4 = 100 μm 7H.1-4 = 50 μm

**Supplemental Video 1: Three dimensional features of whole ANG.** (0:00) 3D transparency projection of whole ANG captured on light sheet microscope seen in Fig. 2A. (0:03) 3D maximum intensity projection. (0:07) Tubules within the dorsal 1/3 of ANG are disorganized and cross over medial plane. (0:11) Tubules within the middle of the ANG display bilateral symmetry on both sides of the medial plane (red plane). (0:17) Individually traced tubules of the whole ANG. (0:28) Traced tubules superimposed onto 3D transparency projection.

**Supplemental Video 2: Tubule convergence underneath NG.** (0:00) 3D volume projection of quadrant of ANG seen in Fig. 2F. (0:04) Inter-gland space highlighted in yellow. (0:13) The inter-gland space is lined with pores at which ANG tubules terminate. (0:18) Individually traced tubules converge at point underneath the NG. Sectioning into tissue illustrates tracing of ANG tubules. (0:31) Some converging tubules weave back and forth through imaging planes. (0:37) 3D maximum intensity projection of ANG quadrant, as seen in Fig. 2G.

**Supplemental Video 3: The inter-gland space in 3D.** (0:00) 3D volume projection of quadrant of ANG seen in Fig. 2F. Inter-gland space is highlighted in purple. (0:06) Segmenting into tissue to reveal inter-gland space between ANG and NG. (0:12) The inter-gland space is lined with pores at which ANG tubules terminate. (0:20) The inter-gland space is highlighted in purple; orientation and segmentation is as seen in Fig. 3C.

**Supplemental Video 4: Visualizing Bacterial Deposition from ANG.** Video showing small debris spinning in the inter-gland space between the NG (top left) and ANG. Peristaltic motion of ANG tubules appears to push tubule contents towards the inter-gland space.
